# CD4^+^CD25^+^ T Cells in primary malignant hypertension related kidney injury

**DOI:** 10.1038/srep27659

**Published:** 2016-06-09

**Authors:** Hongdong Huang, Yang Luo, Yumei Liang, Xidai Long, Youming Peng, Zhihua Liu, Xiaojun Wen, Meng Jia, Ru Tian, Chengli Bai, Cui Li, Fuliang He, Qiushi Lin, Xueyan Wang, Xiaoqun Dong

**Affiliations:** 1 Division of Nephrology, Beijing Shijitan Hospital, Capital Medical University, The 9th Affiliated Hospital of Peking University, Beijing, P.R. China; 2 Division of Nephrology, Hunan Normal University, Hunan Provincial People’s Hospital of China, Changsha, Hunan Province, P.R. China; 3 Department of Liver Surgery, the Affiliated Renji Hospital, Shanghai Jiao Tong University School of Medicine, Shanghai, P.R. China; 4 Hunan Key Laboratory of Nephrology and Hemoperfusion, Division of Nephrology, Second Xiangya Hospital of Central South University, Changsha, Hunan Province, P.R. China; 5 Department of Interventional Therapy, Beijing Shijitan Hospital, Capital Medical University, The 9th Affiliated Hospital of Peking University, Beijing, P.R. China; 6 Section of Hematology/Oncology, Section of Gastroenterology, Stephenson Cancer Center, Department of Internal Medicine, College of Medicine, The University of Oklahoma Health Sciences Center, USA; 7 Center of Allergy, Beijing Shijitan Hospital, Capital Medical University, Beijing, P.R. China

## Abstract

CD4^+^CD25^+^ T cells are critical for maintenance of immunologic self-tolerance. We measured the number of CD4^+^CD25^+^ cells in the patients with primary malignant hypertension related kidney injury, to explore the molecular pathogenesis of this disease. We selected 30 patients with primary malignant hypertension related kidney injury and 30 healthy volunteers. Information on clinical characteristics and laboratory tests was obtained from each subject. The number of CD4^+^CD25^+^ cells and glomerular injury were assessed by flow cytometry and histopathology, respectively. Both serum IL-2, IL-4, and IL-6 and endothelial cell markers were analyzed by ELISA. ADAMTS13 antibody was detected by Western blotting. CD4^+^CD25^+^ cells were significantly reduced in patients with primary malignant hypertension related kidney injury compared to controls (*P* < 0.05). The number of CD4^+^CD25^+^ cells was negatively related to blood urea nitrogen, serum uric acid, proteinuria, and supernatant IL-4; whereas positively associated with estimated glomerular filtration rate in patients. Gradually decreasing CD4^+^CD25^+^ cells were also found as increasing renal injury. Additionally, patients exhibited increasing supernatant IL-4, serum IL-2 and IL-6, endothelial cell markers, and anti-ADAMTS13 antibody compared with controls (all *P* < 0.05). CD4^+^CD25^+^ cells may play a key role in the pathogenesis of primary malignant hypertension related kidney injury.

Malignant hypertension (MHTN) is defined as severely elevated blood pressure (BP) (diastolic BP > 130 mm Hg) with a Keith and Wagener stage III or IV retinopathy^1^. MHTN is a rare but serious syndrome accounting for 1% of essential hypertension, which can cause neurological, renal and cardiac complications. Despite improved anti-hypertensive medication, the incidence of MHTN fails to decline^2^. MHTN is characterized by severe hypertension and acute multi-organ ischemic complications including thrombotic microangiopathy (TMA). Renal TMA accompanying MHTN is due to thrombosis of small vessels, intravascular hemolysis with red cell fragments (Schistocytes), and platelet consumption^3^.

The vascular injury observed in MHTN is unique, where fibrinoid necrosis and proliferative endarteritis affect resistance vessels. Fibrinoid necrosis is defined as vascular smooth muscle cell death with deposition of plasma proteins (e.g., fibrin). Proliferative endarteritis is defined as chronic endarteritis accompanied by a marked increase of fibrous tissue in the inner lining of the artery, characterized by myointimal proliferation with luminal narrowing. However, the triggers and molecular mechanisms involved in these vessel pathologies remain unclear, although several reports indicate the involvement of inflammatory mediators^4^,^5^.

Recent studies have shown that CD4^**+**^CD25^**+**^ cells display a crucial role in the immunological self-tolerance via inhibiting the proliferation and activation of autoreactive T cells^6^. Their depletion might result in the organ-specific autoimmunity developing and autoimmunity diseases, and these changes could be prevented by CD4^**+**^CD25^**+**^ cells in the animal models^7^. Powrie and colleagues^8^ demonstrated that transfer of CD4^**+**^CD25^**+**^ cells could not only protect the mice from developing inflammatory bowel disease, but also reverse the established gastrointestinal inflammation. Until now, CD4^**+**^CD25^**+**^ cells have shown to be regulators in almost all of allergic diseases, transplant rejection, and animal models with human organ specific diseases^9^.

Regulatory T lymphocytes (Tregs) can regulate or suppress the function of other immune cells. CD4^**+**^CD25^**+**^ cells, a type of Tregs, have been identified in mice and humans as a distinct population of CD4^+^ T cells that constitutively express the interleukin (IL)-2 receptor α-chain (CD25)^10^,^11^. CD4^**+**^CD25^**+**^ cells have been shown to dampen local anti-tumor responses and to prevent sterilizing immunity against certain chronic infectious agents. CD4^**+**^CD25^**+**^ cells occasionally mediate peripheral tolerance, leading to an exacerbated inflammatory/allergic reaction or autoimmunity^12^.

To date, there are very few studies of CD4^**+**^CD25^**+**^ cells in human MHTN related kidney injury. Here, we investigated the association between the number change of CD4^**+**^CD25^**+**^ cells and the development of MHTN related kidney injury using a case-control study to test the hypothesis that a numerical or functional deficit of CD4^**+**^CD25^**+**^ cells might trigger the development of disease.

## Methods

### Subjects

Thirty patients with MHTN related kidney injury were included in this study. The patients were diagnosed with clinically and renal biopsy confirmed TMA, hospitalized in Hunan Provincial People’s Hospital and Second Xiangya Hospital of Central South University from January 2006 to February 2014. The patients included 20 men and 10 women, aged 25–57 years (mean ± SD: 42.5 ± 8.61 years). All patients met the criteria for MHTN related kidney injury^13^. Kidney injury was defined as renal damages and/or functional impairment. Secondary MHTN (e.g., renovascular hypertension, primary hyperaldosteronism, pheochromocytoma, polyarteritis nodosa, and renal parenchymal disorders) was excluded. Other factors that could lead to TMA, including cyclosporine A toxicity, liver cirrhosis, cancer, and postpartum renal failure, were also excluded.

Thirty healthy volunteers including 19 men and 11 women, age 30–56 years; (mean ± SD: 43.7 ± 9.65 years) were selected as controls. Patients and controls were all Chinese Han and matched by age and gender.

The present study was approved by the Ethical Committee of Hunan Provincial People’s Hospital and the Second Xiang-ya Hospital of Central South University. All the experiments were carried out in accordance with the approved guidelines and regulations. Each subject had signed written informed consent.

### Calculation of estimated glomerular filtration rate (eGFR)

Renal function was assessed by eGFR, which was calculated using CKD-EPI (Chronic Kidney Disease Epidemiology Collaboration) creatinine equation^14^. eGFR = 141 X min(Scr/κ,1)^α^ X max(Scr/κ,1)^−1.209^ × 0.993^Age^ X 1.018 [if female], where Scr. is serum creatinine (mg/dL), κ is 0.7 for females and 0.9 for males, respectively, α is −0.329 for females and −0.411 for males, respectively, min indicates the minimum of Scr/κ or 1, and max indicates the maximum of Scr/κ or 1.

### Data Collection

After informed consent was obtained, peripheral blood and urine samples and clinical data were collected. None of the patients had undergone treatment with angiotensin converting enzyme (ACE) inhibitor, immunosuppressive agents, steroid, or AT1 receptor blockers before clinical samples collection. The laboratory examinations included urinalysis, complete blood count, serum chemistries, endothelial markers [von Willebrand factor (vWF), vascular cell adhesion molecule (VCAM)], and immunologic serologic index (anti-nuclear antibody, anti–double-stranded DNA antibody, anti-neutrophil cytoplasmic antibody, anti -endothelial cell antibody, and complement components C3).

### Flow cytometry

The live-dead lymphocytes and CD4^+^CD25^+^ cells in the peripheral blood were evaluated according to previously published flow cytometry^15^. Briefly, for peripheral blood live-dead lymphocyte assay, blood was mixed and incubated with 10 μL Propidium iodide for 10 min at room temperature, then analyzed by flow cytometry. The analysis and gates were restricted to lymphocytes. For peripheral blood CD4^+^CD25^+^ cells, blood was mixed and incubated with 10 μL monoclonal FITC-labeled anti-human CD4 and PE-anti-CD25 for 30 min at room temperature. After that, the samples were fixed and analyzed by flow cytometry. The analysis and gates were restricted to live lymphocytes. All experiments were performed in triplicate and repeated three times.

### Isolation and culture of peripheral blood mononuclear cells (PBMCs)

PBMCs were isolated and cultured according to previously published methods^15^. In brief, lymphocyte Separation Medium (Flow Labs, McLean, VA) in combination with density gradient centrifugation was used to isolate PBMCs from heparinized peripheral blood. Cells were next cultured in RPMI1640 medium containing 10% fetal bovine serum (HyClone) and 20 μg/ml phytohaemagglutinin (Sigma) in an atmosphere of 5% CO_2_ at 37 °C. After twenty-four hours, cell-free supernatant was obtained and frozen at −70 °C until assayed.

### Enzyme-linked immunosorbent assay (ELISA)

The cell-free supernatant IL-4, serum IL-2, IL-6, and endothelial cell markers were tested using ELISA kits (R&D Systems, USA) according to the manufacturer’s instruction. All experiments were performed in triplicate and repeated three times.

### Western Blotting for ADAMTS13 antibody

The serum of producing recombinant ADAMTS13 antibody^16^ (100 ng/lane) was added to gel-loading buffer (50 mM Tris-HCl pH 6.8, 2% sodium dodecyl sulphate, 0.1% bromophenol blue, 10% glycerol). Next, 7% sodium dodecyl sulphate polyacryl gel electrophoresis was performed in Tris-glycine buffer (pH 8.3). Migrated samples were transferred onto a pure nitrocellulose membrane (Bio-Rad, Hercules, CA, USA). After blocking with skimmed milk, membranes were incubated with citrated plasma samples (diluted 1:100 in TBS, 5% skimmed milk, 0.05% Tween 20, pH 7.4) used as a possible source of anti-ADAMTS13 antibody (R&D Systems, USA). Bound antibodies were visualized using alkaline phosphatase-labeled anti-human immunoglobulin (diluted 1:2000) and an alkaline phosphatase conjugate substrate kit (AmershamBioscences, Uppsala, Sweden). The β-actin was probed as a control for equal protein loading. Experiments were repeated three times; each experiment was in triplicate.

### Renal histopathology examination

Kidney injury was evaluated using histopathological methods according to previously published methods^17^. Briefly, kidney biopsy samples from 30 patients with primary MHTN related kidney injury were fixed (using 10% buffered formalin), dehydrated, embedded in paraffin, and examined in the slide via the hematoxylin and eosin staining, periodic acid-schiff, or immunofluorescence techniques. Renal histological and immunofluorescence results, including chronicity indices (CI), activity indices (AI), complement component C3, IgG, IgA, and IgM, were graded semi-quantitatively on a mild, moderate, and marked scale (also called 1+, 2+, and 3+ scale) as previously described^18^,^19^,^20^. In this study, CI consists of glomerular sclerosis, fibrous crescents, renal tubular atrophy and interstitial fibrosis; whereas AI was defined according to glomerular hypercellularity, leucocyte exudation, fibrinoid necrosis, cellular crescents, hyaline deposits and interstitial inflammation.

### Statistical analysis

All statistical analyses were performed using Statistical Package for Social Sciences version 16.0 (SPSS Inc., USA) and Graph Pad Prism 5.0 (Graph Pad Inc., USA). Data were checked for normality of distribution using the Kolmogorov-Smirnov test and expressed as mean ± standard deviation (SD) or median and interquartile range. Differences between the two groups were analyzed using *t* -test or Mann-Whitney *U*-test. The Spearman *r* test was used to analyze the relationships between different values. A *p* value < 0.05 was considered statistically significant.

## Results

### Flow cytometry analysis of peripheral blood live-dead lymphocytes

**Figure 1:**
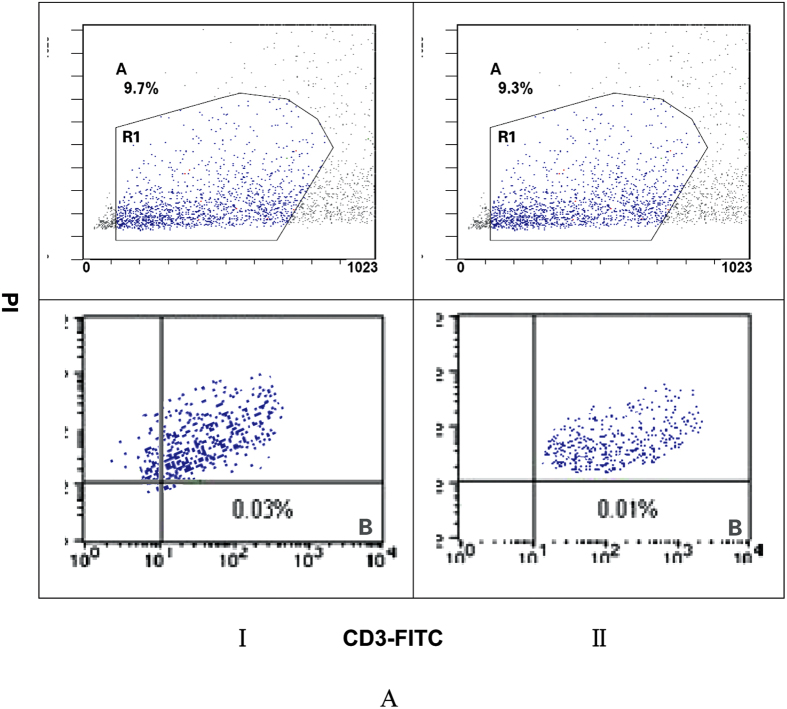
Live-dead lymphocytes in peripheral blood. Lymphocytes were counted using the indicated gates R1 by flow cytometry. (**B**) Dead lymphocytes; I: patients with primary malignant hypertension related kidney injury; II: Controls. Live, Dead lymphocytes respectively were not significantly different in between patients and controls (all *P* > 0.05).

The results from flow cytometry analysis peripheral blood live-dead lymphocytes showed live lymphocytes accounted for 99% in both patients with MHTN related kidney injury and controls (CD3- FITC positive cells; PI negative cells); whereas the number of dead lymphocytes was less than 1% (Fig. 1). There were no differences between patients and controls in terms of the distribution of Live and Dead lymphocytes (all *P* > 0.05).

### Flow cytometry analysis of CD4^+^CD25^+^ cells

**Figure 2:**
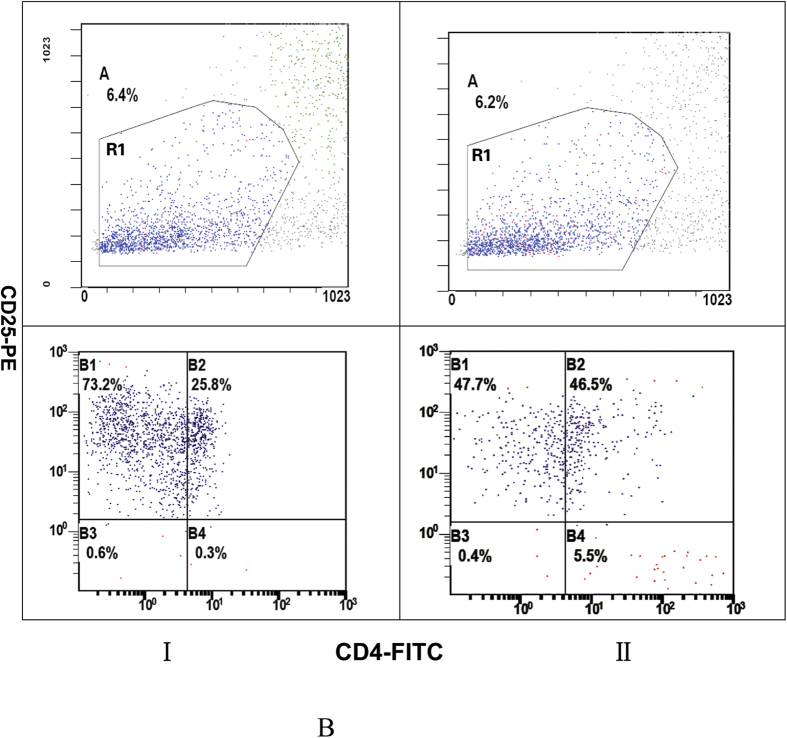
CD4^+^CD25^+^ cells in peripheral blood. CD4^+^CD25^+^ cells were counted using the indicated gates by flow cytometry. B4: CD4^+^CD25^+^cells; I: patients with primary malignant hypertension related kidney injury; II: Controls. CD4^+^CD25^+^ cells dramaticlly decreased in patients compared to controls (*P* < 0.05).

We next analyzed the population of CD4^**+**^CD25^**+**^ cells in peripheral blood using the flow cytometry (Fig. 2). The results exhibited that the number of CD4^**+**^CD25^**+**^ cells was significantly decreased in patients with primary MHTN related kidney injury compared to controls (*P* < 0.05) (Table 1).

### Clinical and histopathological findings in patients

**Figure 3:**
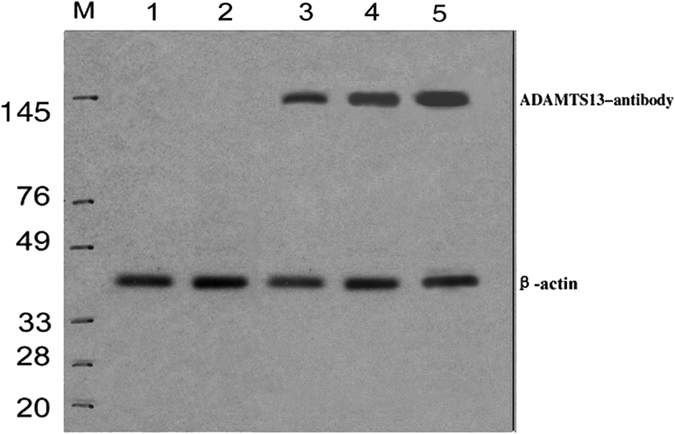
Serum ADAMTS13 antibody by Western blotting. ADAMTS13 antibody (positive %) accounted for 81% of the primary MHTN related kidney injury patients. Controls were negative. Lane M: Marker; lane 1: Controls; lane 2: Controls; lane 3: primary MHTN related kidney injury patients (Mild lesion); lane 4: primary MHTN related kidney injury patients (Moderate lesion); lane 5: primary MHTN related kidney injury patients(Marked lesion).

**Figure 4:**
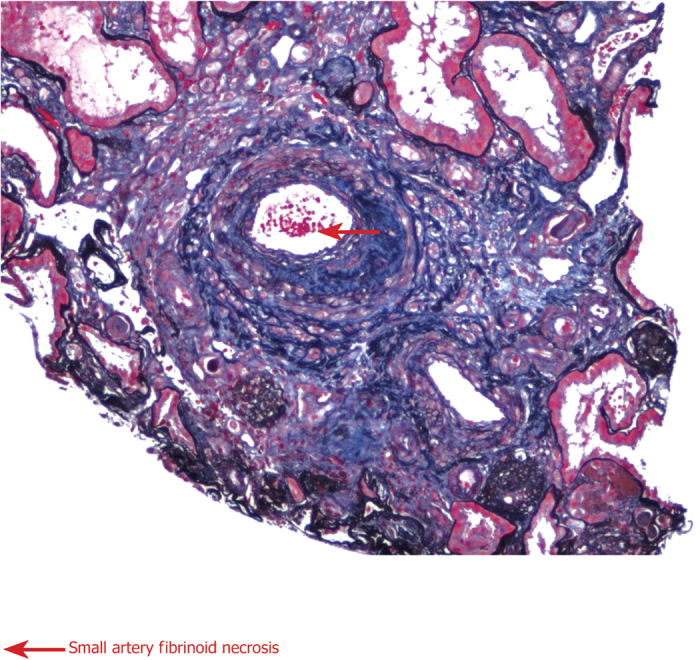
Histopathological findings in primary malignant hypertension related kidney injury (40×). Endothelial injury and thrombogenesis precedes myointimal swelling and proliferation, leading to vascular lumina narrowing or obliteration.

**Figure 5:**
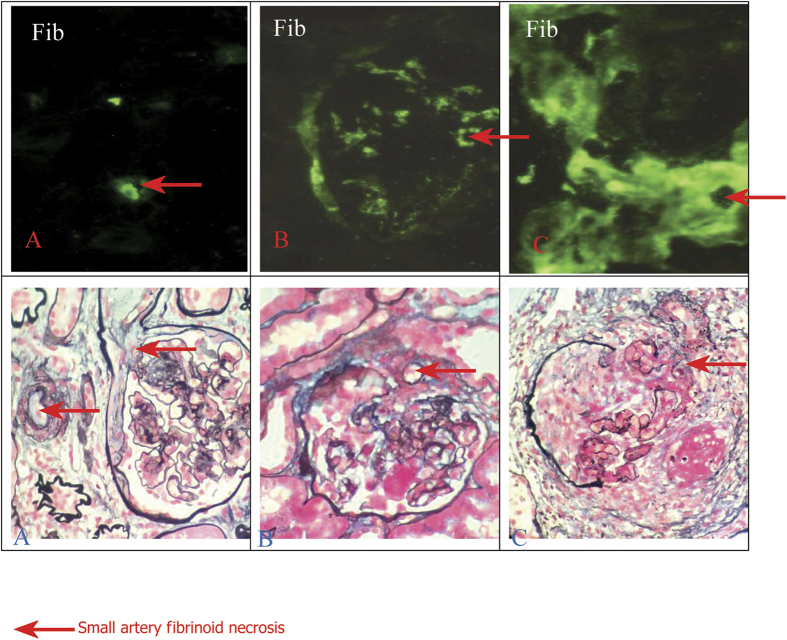
Histopathological findings in primary malignant hypertension related kidney injury (40×). (**A**) Mild lesion; (**B**) Moderate lesion; and (**C**) Marked lesion.

Table 2 summarizes clinical and histopathological findings in patients with MHTN related kidney injury. Serum IL-2, IL-6, supernatant IL-4, endothelial cell markers, ADAMTS13 antibody (Fig. 3), VCAM and vWF dramaticlly increased in patients compared to controls (^*^P < 0.05). Endothelial injury manifests with swelling and detachment of endothelial cells from the basement membrane, expansion of the subendothelial space, and newly formed basement membrane-like material. In arterioles, endothelial injury precedes myointimal swelling and proliferation, leading to vascular lumina narrowing or obliteration, secondary glomerular ischemia, which is characterized by glomerular tuft collapse, garland-like wrinkling and thickening of the capillary wall. Endothelial cell injury is likely the common determinant of a cascade of events that lead to irreversible renal failure (Figs 4 and 5)^21^.

### Number of CD4^+^CD25^+^ cells and clinical parameters

The association between the number of CD4^**+**^CD25^**+**^ cells and clinical parameters were examined in patients with primary MHTN related kidney injury (Table 3). The number of CD4^**+**^CD25^**+**^ cells was negatively correlated with the level of serum blood urea nitrogen (BUN) or uric acid, whereas positively correlated with eGFR (*P < 0.05). These results indicate that the number of CD4^**+**^CD25^**+**^ cells reflects renal function. In addition, the number of CD4^**+**^CD25^**+**^ cells were negatively correlated with the level of urine protein or supernatant IL-4 in patients (*P < 0.05).

### Correlation between the number of CD4^+^CD25^+^ cells and histological classification of primary MHTN related kidney injury

**Figure 6:**
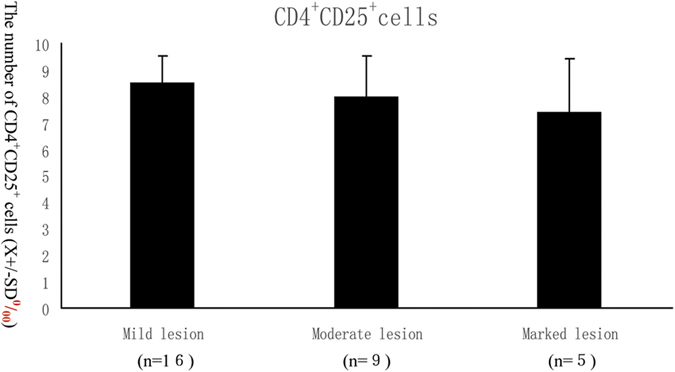
The number of CD4^+^CD25^+^ cells and severity of disease. The number of CD4^+^CD25^+^ cells in primary MHTN related kidney injury tended to decrease in parallel with the severity of the lesion increased, although the difference was not significant.

The number of CD4^**+**^CD25^**+**^ cells in individualls with MHTN related kidney injury tended to decrease in parallel with increased the histological classification degrees of nephropathy, as determined by the classification^19^ (Fig. 5), however, the difference was not significant (Fig. 6).

## Discussion

The pathogenesis of primary MHTN related kidney injury is unclear, although some factors such as direct pressure effects (forced dilatation), excessive vasoconstriction, volume depletion, endocrine/paracrine renin–angiotens, nitricoxide, natriuretic peptides, endothelins and oxidative stress might be related to the process^22^,^23^,^24^.

The CD4^**+**^CD25^**+**^ cells, a kind of important immunological modulatory cells, can reduce cytokine production and limit the development of a proinflammatory CD4^+^ Th2 phenotype^25^. The functionary deficiency or suppression of CD4^+^CD25^+^ cells might result in the inappropriate balance between allergen activation of CD4^+^CD25^+^ cells and effector Th2 cells^26^,^27^,^28^. This type of cells also plays a key role in immunomodulatory process following antigen inhalation^29^. Th1 responses (releasing IL-2, IFNγ, and TNF-β) are more prone to be regulated by CD4^**+**^CD25^**+**^ T cells than Th2 responses (releasing IL-4, IL-5, IL-6, IL-10, and IL-13)^30^. Our previous studies^15^,^31^,^32^ have shown that tonsillar CD4^**+**^CD25^**+**^ cells were significantly decreased in IgA nephropathy, multiple myeloma related renal impairment as well as thrombotic thrombocytopenic purpura (TTP) associated with systemic lupus erythematosus (SLE). Furthermore, we have found that tonsillar CD4^**+**^CD25^**+**^ cells from IgA nephropathy patients present reduced immunosuppressive activity in experimental IgA nephropathy rats^33^.

In the current study, CD4^**+**^CD25^**+**^ cells significantly decreased in patients with primary MHTN related kidney injury. Supernatant IL-4, serum IL-2 and IL-6, endothelial cell markers, ADAMTS13-antibody, VCAM, vWF, and urine protein and erythrocytes significantly elevated in those patients. The number of CD4^**+**^CD25^**+**^ cells was negatively related to serum BUN, uric acid and urinary protein, and supernatant IL-4; whereas positively related to eGFR. Furthermore, with increasing severity of histologically assessed nephropathy, the number of CD4^**+**^CD25^**+**^ cells tended to gradually decrease. However, it is unclear whether the decrease of this type cells is the cause or the consequence of the disease.

When patients with primary MHTN related kidney injury have decreased CD4^**+**^CD25^**+**^ cells, their lymphocytes would react significantly more strongly to antigens, leading to higher levels of cytokine (IL-2, IL-4, and IL-6) production. Excessive cytokine production in turn would enhance the production of antibodies against endothelial cell markers or ADAMTS13 antibody. Therefore, high levels of cytokine and antibodies are present in the blood, which directly damage vascular endothelium, as demonstrated by elevated levels of endothelial functional parameters vWF and VCAM. These changes lead to platelet abnormalities, complement activation or imbalance between blood coagulation and plasminogen, and thus contribute to the development of TMA, ischemia and damage^34^,^35^,^36^,^37^,^38^.

ADAMTS13 is a zinc-containing enzyme that cleaves large VWF multimers. Congenital or acquired deficiency of ADAMTS13 can increase their thrombogenicity. Reduced ADAMTS13 activity has been associated with TMA in MHTN^39^. We also detected elevated levels of anti-ADAMTS13 and anti-vWF antibodies in MHTN related kidney injury.

To sum up, CD4^**+**^CD25^**+**^ cells might play an important role in the pathogenesis of primary MHTN related kidney injury. The number of CD4^**+**^CD25^**+**^ cells tended to gradually decrease with increasing severity of histologically assessed nephropathy. Additional studies are necessary to demonstrate functional deficits or other biological properties of CD4^**+**^CD25^**+**^ cells in primary MHTN related kidney injury.

## Additional Information

**How to cite this article**: Huang, H. *et al.* CD4^+^CD25^+^ T Cells in primary malignant hypertension related kidney injury. *Sci. Rep.* **6**, 27659; doi: 10.1038/srep27659 (2016).

## Figures and Tables

**Table 1:** The number of CD4^+^CD25^+^ cells in peripheral blood (%, mean ± SD).

Index	Patients	Controls
CD4^+^CD25^+^ cells	0.85 ± 0.106^a^	2.93 ± 0.207

^a^*P* < 0.05.

**Table 2:** Clinical and histopathological findings in patients and controls.

Index (mean ± SD)	Patients	Controls
BP (systolic/diastolic)	187.5 ± 16.3^*^/140 ± 7.2^*^	124 ± 11.5/73 ± 9.8
Proteinuria (g/day)	1.25 ± 0.31	–
Hematuria (×10^4^ cells/mL)	364.75 ± 2.18	–
BUN (mg/dL)	28.7 ± 0.75^*^	12.5 ± 0.13
Serum Cr. (mg/dL)	6.83 ± 0.25^*^	0.58 ± 0.12
Serum uric acid (μmol/L)	467.62 ± 73.81^*^	246.37 ± 28.34
eGFR (mL/min/1.73 m^2^)	67.12 ± 10.23^*^	101.32 ± 7.41
Serum C3 (mg/dL)	119.5 ± 15.4	121.5 ± 13.7
Serum IL-2 (pg/ml)	104.6 ± 16.3^*^	24.7 ± 15.2
Supernatant IL-4 (pg/ml)	536 ± 24.03^*^	213.46 ± 35.67
Serum IL-6 (pg/ml)	60.21 ± 12.41^*^	30.43 ± 11.42
AI/CI (median)	10.4 ± 2.1/3.6 ± 0.5	–
vWF (%)	215.73 ± 12.40	–
VCAM (ng/mL)	1135.26 ± 143.87	–
AECA (positive %)	72	–
ADAMTS13-antibody (positive %)	81	–

BP, Blood pressure; eGFR, estimated glomerular filtration rate; AI, activity index; CI, chronicity index; vWF, von Wille brand factor; VCAM, vascular cell adhesion molecule; AECA, anti -endothelial cell antibody.

^*^P < 0.05 compared to controls.

**Table 3:** Frequency of CD4^+^CD25^+^ cells (%) and clinical parameters in patients.

Index	r	*p*
SBP	−0.236	n.s.
DBP	−0.310	n.s.
BUN	−0.524	<0.05
SUA	−0.538	<0.01
eGFR	0.867	<0.01
24 h UP	−0.583	<0.01
Hematuria	−0.206	n.s.
Serum C3	0.233	n.s.
Serum IL-2	−0.124	n.s.
Supernatant IL-4	−0.746	<0.01
Serum IL-6	−0.228	n.s.
vWF (%)	−0.231	n.s.
VCAM (ng/mL)	−0.185	n.s.

n.s., not significant; SBP, systolic blood pressure; DBP, diastolic blood pressure; Scr, serum creatinine; SUA, serum uric acid; eGFR, estimated glomerular filtration rate; 24-h UP, 24- hours urinary protein. vWF, von Willebrand factor; VCAM, vascular cell adhesion molecule.
